# Putative Neurostructural Phenotypes of Genetic Risk for Alzheimer's Disease in Youth with Bipolar Disorder

**DOI:** 10.1002/alz70856_099327

**Published:** 2025-12-24

**Authors:** Gabrielle Walach, Kody Kennedy, Mikaela Dimick, Clement Zai, Brad MacIntosh, Benjamin Goldstein

**Affiliations:** ^1^ Centre for Addiction and Mental Health, Toronto, ON, Canada; ^2^ University of Toronto, Toronto, ON, Canada

## Abstract

**Background:**

Bipolar disorder (BD) is linked to an increased risk of Alzheimer's disease (AD). It is well established that the AD process long precedes onset of clinical symptoms, highlighting the importance of understanding the early genesis of AD. Evaluating genetic risk for AD in relation to brain structure in youth may provide insights regarding regional neurostructural susceptibility to AD. The anterior cingulate cortex has been implicated in both BD and in relation to polygenic risk scores (PRS) for AD in healthy children.

**Methods:**

This study investigated AD‐PRS derived from adult studies, both as main effect and interaction with diagnosis, on brain structure using T1‐weighted magnetic resonance imaging data. Participants included 112 youth aged 13‐20 years (*n* = 67 BD; *n* = 45 healthy controls [HC]). AD‐PRS were calculated using PRS‐CS‐auto. General linear models assessed the association between AD‐PRS and regional brain structure, controlling for age, sex, intracranial volume, and the first two genetic principal components. Vertex‐wise analyses and an anterior cingulate cortex region of interest analyses were conducted, as well as sex‐stratified analyses.

**Results:**

In the whole sample, higher AD‐PRS was associated with lower superior temporal gyrus volume (*p* = 0.03). In interaction analyses, higher AD‐PRS was associated with lower regional structure to a greater extent in the BD vs. HC group, including: superior temporal gyrus (*p* = 0.008) and precuneus (*p* = 0.01) volume, and rostral middle frontal (L: *p* <0.001; R: *p* = 0.03), caudal middle frontal (*p* = 0.001), superior frontal (*p* = 0.005), precentral (*p* = 0.049) gyri, and superior parietal lobule (*p* = 0.04) thickness. In sex‐stratified analyses, higher AD‐PRS was associated with lower superior parietal lobule surface area (*p* = 0.03) and volume (*p* <0.001) in females. In males, higher AD‐PRS was associated with higher cortical thickness and volume in the supramarginal and rostral middle frontal gyri, respectively (*p* <0.001; *p* = 0.004). Region of interest analyses were not significant.

**Conclusion:**

Higher AD‐PRS is associated with smaller brain structure metrics in youth with BD compared to HC. Findings also highlight potential sex differences. Future longitudinal studies should be conducted to investigate dynamic changes in the association between AD‐PRS and brain structure across development.

